# A Non-Contact Electrostatic Potential Sensor Based on Cantilever Micro-Vibration for Surface Potential Measurement of Insulating Components

**DOI:** 10.3390/s26020362

**Published:** 2026-01-06

**Authors:** Chen Chen, Ruitong Zhou, Yutong Zhang, Yang Li, Qingyu Wang, Peng Liu, Zongren Peng

**Affiliations:** School of Electrical Engineering, Xi’an Jiaotong University, Xi’an 710049, China; zrtdqyxx@stu.xjtu.edu.cn (R.Z.); zhangyt6517@163.com (Y.Z.); liyang3121@stu.xjtu.edu.cn (Y.L.); wqy.1991624@xjtu.edu.cn (Q.W.); pengliu@xjtu.edu.cn (P.L.); zrpeng@mail.xjtu.edu.cn (Z.P.)

**Keywords:** surface potential measurement, HVDC power equipment, electrostatic potential sensor, cantilever micro-vibration modulation, insulator surface electric field

## Abstract

With the rapid development of high-voltage DC (HVDC) power systems, accurate measurement of surface electrostatic potential on insulating components has become critical for electric field assessment and insulation reliability. This paper proposes an electrostatic potential sensor based on cantilever micro-vibration modulation, which employs piezoelectric actuators to drive high-frequency micro-vibration of cantilever-type shielding electrodes, converting the static electrostatic potential into an alternating induced charge signal. An electrostatic induction model is established to describe the sensing principle, and the influence of structural and operating parameters on sensitivity is analyzed. Multi-physics coupled simulations are conducted to optimize the cantilever geometry and modulation frequency, aiming to enhance modulation efficiency while maintaining a compact sensor structure. To validate the effectiveness of the proposed sensor, an electrostatic potential measurement platform for insulating components is constructed, obtaining response curves of the sensor at different potentials and establishing a compensation model for the working distance correction coefficient. The experimental results demonstrate that the sensor achieves a maximum measurement error of 0.92% and a linearity of 0.47% within the 1–10 kV range. Surface potential distribution measurements of a post insulator under DC voltage agreed well with simulation results, demonstrating the effectiveness and applicability of the proposed sensor for HVDC insulation monitoring.

## 1. Introduction

With the development of long-distance, high-capacity power transmission, voltage levels in high-voltage direct current (HVDC) systems continue to increase, making charge accumulation at the gas–solid interfaces of insulating materials an increasingly critical issue [1,2]. Non-uniform surface charge distributions on power equipment insulation surfaces can significantly enhance local electric field intensity and, through charge–field–material coupling effects [3,4], reduce surface flashover voltage, thereby increasing the risk of insulation degradation and electrical breakdown in HVDC power apparatus [5]. Consequently, surface charge management has become a central challenge in DC insulation research. Accurate measurement of surface electrostatic potential on power equipment is therefore essential for investigating charge migration mechanisms [6] and constructing multi-physics coupling models [7]. This is crucial for ensuring the long-term reliability of high-voltage DC grids.

Electrostatic potential sensing, as a primary means to characterize surface charge distributions on insulators, can be broadly classified into optical and electrical approaches. Optical methods exploit the Pockels effect in electro-optic crystals to infer surface charge distributions from electric-field-induced refractive index variations [8,9]. However, these methods are typically contact-based [10,11], and in non-contact configurations, their DC response degrades due to depolarization at crystal interfaces, requiring rotating modulation for signal demodulation [12,13]. Such mechanical modulation increases sensor size and introduces vibration-induced noise, which restricts their practical deployment near energized power equipment.

Electrical sensing approaches rely on electrostatic induction and are generally categorized as passive or active. Passive probes commonly employ capacitor structures with grounded shields and capacitive voltage division principles to convert the induced electrostatic voltage into measurable signals via operational amplifiers [14,15]. Their performance is strongly affected by parasitic capacitances, which cause charge decay and result in poor stability under high-voltage DC conditions [16]. Active probes address this limitation by introducing mechanical modulation to convert static signals into alternating ones [17]. For instance, the field-mill-type electrostatic sensor developed by Beihang University uses motor-driven grounded shield electrodes that periodically expose the sensing electrodes, converting the measured object’s potential into an alternating current signal [18,19]. Nevertheless, the bulky rotating mechanisms of such sensors limit their applicability to compact or spatially constrained power equipment, while their relatively low modulation frequencies constrain sensitivity. To mitigate these issues, vibrating-capacitor-based sensors have been developed, in which electromagnetic or piezoelectric actuation modulates the effective sensing capacitance [20]. MEMS (Micro-Electro-Mechanical Systems)-based implementations further reduce sensor size and enable higher modulation frequencies [21], but challenges such as thermal noise and localized high-voltage discharge still constrain measurement accuracy and long-term reliability [22,23]. Kelvin probe-based sensors are the most commonly adopted tools in surface charge, which offer high potential resolution and high spatial resolution. However, their operating principle requires very short stand-off distances, which significantly restricts their applicability in high-voltage DC power equipment.

Despite these advances, existing electrostatic potential sensing techniques remain constrained by a fundamental trade-off between sensor compactness, modulation efficiency, and measurement accuracy when applied to surface potential measurements of power equipment under DC electric fields. In practical applications, sensors must operate at a sufficient distance from energized surfaces to avoid discharge risks while minimizing disturbance to the original electric field. These requirements impose strict constraints on sensor size, modulation efficiency, and sensitivity, indicating that further improvements are necessary to meet the application-specific demands of HVDC power equipment diagnostics.

To address these challenges, this paper proposes a non-contact electrostatic potential sensor based on cantilever micro-vibration modulation. The sensor employs piezoelectric ceramics to drive controlled, high-frequency cantilever vibrations, enabling efficient modulation of the induced electrostatic signal while maintaining a compact sensor footprint. An electrostatic induction model is established to elucidate the sensing mechanism and derive the sensing equations. To satisfy the requirements of miniaturization and high sensitivity, multi-physics simulations are conducted to analyze cantilever vibration modes, identify key parameters influencing sensor performance, and guide structural optimization. Furthermore, the relationship between the measured electrostatic field and the surface potential is investigated, and a distance-dependent correction model is established to enable accurate surface potential reconstruction under varying probe-to-equipment distances. To validate the effectiveness of the proposed sensor, surface potential distribution measurements were conducted on epoxy insulators. Experimental results demonstrate that the sensor can effectively capture the surface potential distribution of the insulator, with the measured data showing high consistency with theoretical analysis, thus confirming the sensor’s validity.

## 2. Principle and Sensor Design

### 2.1. Operating Principle and Theoretical Model

**Figure 1:**
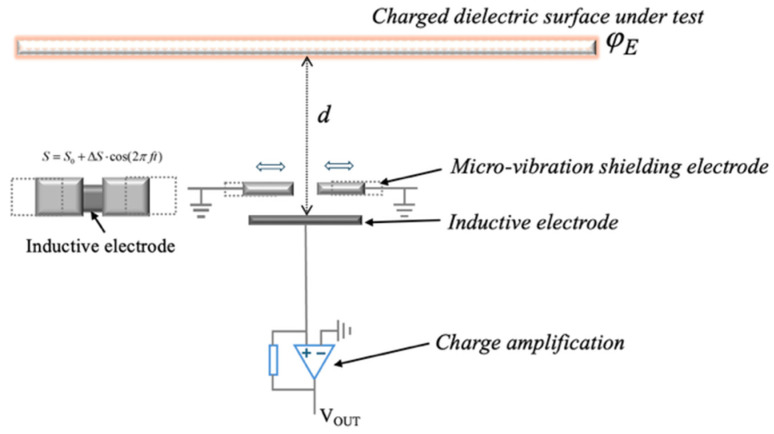
Sensing principle.

The operating principle of the electrostatic potential sensor proposed in this paper is illustrated in Figure 1. The sensor probe mainly consists of an inductive sensing electrode and a micro-vibration shielding electrode. When the sensor probe is positioned in proximity to the surface of an insulating component, such as a DC insulator or spacer, electrostatic induction occurs due to the external electric field generated by surface charges on the equipment. As a result, induced charges are formed on the sensing electrode.

The magnitude of induced charge is determined by the distance between the inductive electrode and the surface of the object being tested, as well as the effective area and dielectric constant. As the shielding electrode undergoes periodic motion under the modulation signal, the induced charge on the inductive electrode changes, generating a current in the circuit. By measuring this current, the electrostatic potential of the charged dielectric surface can be determined.

The distance between the inductive electrode and the charged dielectric surface to be measured is denoted as *d*. In the absence of shielding electrode modulation, the effective area of the inductive electrode is *S*_0_. When the shielding electrode vibrates periodically, the maximum variation in the effective sensing area is defined as Δ*S*. The mechanical modulation of the vibrating shielding electrode can be approximated as sinusoidal motion with a frequency *f*, such that the effective area of the inductive electrode is given by:

(1) $$S=S_{0}+\Delta S\cos(\omega t)$$

where *ω* is the angular frequency of the micro-vibrating shielding electrode, defined as 2*πf*.

Accordingly, the equivalent capacitance between the inductive electrode and the charged dielectric surface is expressed as:

(2) $$C=\frac{\varepsilon (S_{0}+\Delta S\cos\omega t)}{d}$$

where *ε* denotes the dielectric constant of air.

When the sensor probe is exposed to the electrostatic field generated by the surface charges on the power equipment, the induced charge on the sensing electrode is given by

(3) $$Q=C\Delta U=C(\varphi_{E}-V_{0})$$

where *φ_E_* is the surface potential of the measured equipment, and *V*_0_ is the potential of the inductive electrode. The induced charge generates an induced current in the measurement circuit, which can be further expressed as:

(4) $$i=\frac{dQ}{dt}=(\varphi_{E}-V_{0})\frac{dC}{dt}+C\frac{d(\varphi_{E}-V_{0})}{dt}$$

Since *φ_E_* is much larger than *V*_0_, and the potential of the dielectric surface remains approximately constant, the equation simplifies to:

(5) $$i=\varphi_{E}\cdot \frac{dC}{dt}=\varphi_{E}\cdot \frac{\varepsilon }{d}\cdot \Delta S\cdot \omega \cdot \sin\omega t$$

This expression indicates that the sensor output is an alternating current signal whose amplitude is directly related to the surface potential of the power equipment. By extracting the Root Mean Square (RMS) value of this alternating current signal, the potential of the charged dielectric surface can be determined, as expressed by:

(6) $$I=\frac{\varepsilon \Delta S\omega }{\sqrt{2}d}\cdot \varphi_{E}$$

### 2.2. Sensor Design

Based on the above sensing principle, a compact non-contact electrostatic potential sensor dedicated to power equipment surface measurements was developed using cantilever micro-vibration modulation. The sensor adopts a symmetric dual-cantilever shielding electrode configuration driven by piezoelectric ceramic actuators, enabling high-frequency, micrometer-scale mechanical modulation within a confined structure.

Here, piezoelectric ceramic actuators are uniformly bonded to the longitudinal surfaces of the cantilever using high-precision structural adhesive. When an alternating voltage at a designated frequency is applied, the inverse piezoelectric effect induces precisely controlled micrometer-scale deformation, which in turn excites periodic micro-vibration of the cantilever. This vibration dynamically modulates the effective exposure of the sensing electrode to the external electrostatic field, thereby converting a static electrostatic potential into a measurable alternating charge signal.

**Figure 2:**
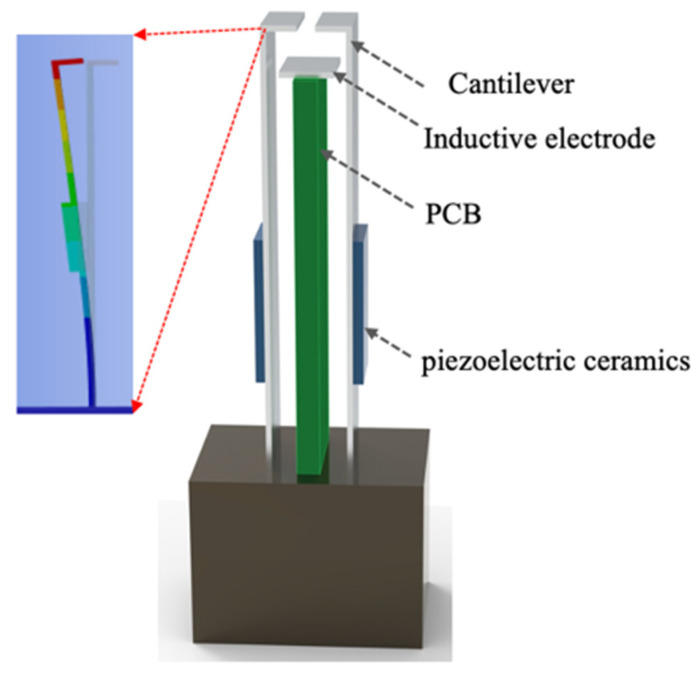
Schematic diagram of the sensor structure.

The overall sensor configuration, shown in Figure 2, integrates a sensing electrode, cantilever-type shielding electrodes, piezoelectric actuators, and a compact printed circuit board (PCB). Here, PZT-5A (Changsha Pengxiang Electronic Technology Co., Ltd., Changsha, China) is employed as the piezoelectric actuators, which shows high piezoelectric excitation efficiency (Detailed information can be found in the Supplementary Materials). The shielding electrodes adopt a distinctive “7-shaped” cantilever geometry to optimize vibration characteristics and modulation efficiency. Dedicated charge amplification and filtering circuits are integrated directly onto the PCB, enabling local demodulation and conditioning of weak induced charge signals and reducing susceptibility to external electromagnetic interference commonly encountered in power environments.

During operation, high-frequency alternating excitation is applied to the piezoelectric ceramics, generating periodic micro-displacement of the cantilever and consequently modulating the effective area of the sensing electrode. The induced charge is converted into a voltage signal through the on-board charge-measurement circuitry, and the RMS value of the output voltage is extracted to quantitatively determine the electrostatic potential of the measured object.

The proposed sensor employs a high-efficiency modulation strategy based on cantilever micro-vibration, which enables high-frequency modulation within a compact structure. Compared with conventional mechanically modulated electrostatic sensors, this approach achieves enhanced sensitivity with a reduced modulation scale, thereby limiting disturbance to the original electric field of the measured power equipment and supporting safe non-contact surface potential measurement at practical distances.

## 3. Structural Optimization

According to the derived sensing Equation (6), the sensitivity of the proposed electrostatic potential sensor is primarily governed by three factors: the effective modulation area of the sensing electrode Δ*S*, the measurement distance *d*, and the modulation frequency *f*. In practical measurements of high-voltage power equipment, the probe–surface distance *d* cannot be excessively reduced due to safety considerations, such as minimizing electric-field disturbance and avoiding electrostatic discharge. Moreover, variations in *d* require corresponding calibration coefficients. Consequently, enhancing sensor sensitivity mainly relies on increasing Δ*S* and vibration frequency *f.*

In the proposed cantilever-based modulation scheme, the effective modulation area Δ*S* is directly determined by the vibration amplitude A of the cantilever. Therefore, improving sensor sensitivity requires systematic optimization of the cantilever’s structural parameters to achieve a large vibration amplitude while maintaining a sufficiently high modulation frequency.

To this end, multi-physics simulations were conducted using ANSYS 2021R1 to analyze the electromechanical response of the cantilever under different structural configurations. For each configuration, sinusoidal excitation voltages with identical amplitude and varying frequency were applied to the piezoelectric ceramic actuators. The resulting vibration amplitude–frequency response of the cantilever was obtained, enabling quantitative evaluation of the modulation performance associated with each structural design.

**Figure 3:**
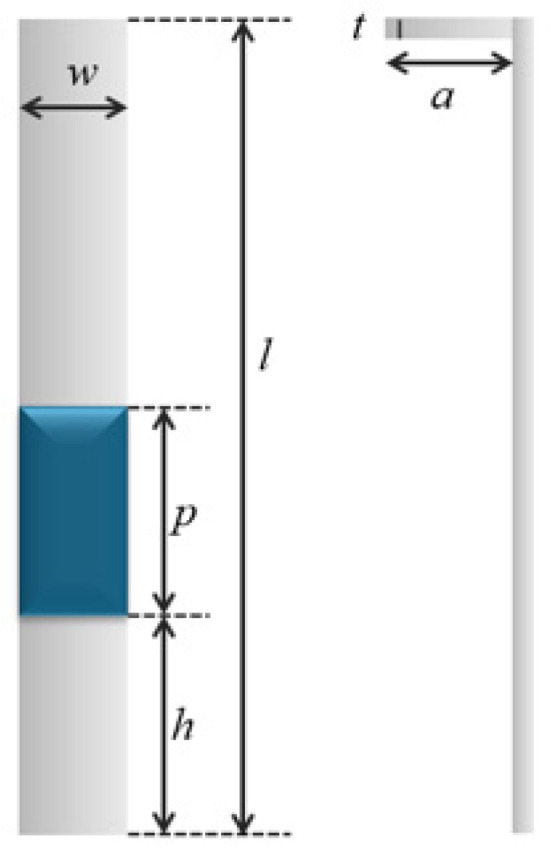
Geometric parameters of the cantilever structure.

The geometric parameters of the cantilever structure are illustrated in Figure 3. The cantilever is characterized by its length *l*, width *w*, and thickness *t*. The piezoelectric ceramic, with length *p*, is bonded along the width direction of the cantilever. The distance from the fixed end of the cantilever to the lower edge of the piezoelectric ceramic is denoted by *h*. The modulation electrode shares the same thickness *t* and has a width *a*.

Parametric sweep simulations indicate that the modulation efficiency of the sensing electrode is predominantly influenced by two structural parameters: the length of the cantilever and the placement position of the piezoelectric ceramic along the cantilever. As a result, the structural optimization in this study focused on these two parameters. For convenience in analysis, a normalized piezoelectric placement parameter is defined as

(7) $$n_{h}=\frac{\left(h+p\right)}{l}$$

where *h* denotes the distance from the fixed end of the cantilever to the center of the piezoelectric ceramic, and *l* represents the total length of the cantilever.

A systematic investigation was performed to evaluate the influence of cantilever length (8 mm≤l≤25 mm) and piezoelectric ceramic position on the dynamic response of the cantilever. Particular attention was given to the coupled relationship between the maximum vibration amplitude *A* and the modulation frequency *f*, as both parameters jointly determine the effective modulation capability. During the simulation, a sinusoidal excitation voltage of 5 V is applied to the piezoelectric ceramic. According to the machining constraints of stainless steel, the baseline parameters are set as follows: cantilever thickness t=0.2 mm, nominal piezoelectric ceramic length of 5 mm, cantilever width of 3 mm, and geometric parameter a=1.5 mm.

**Figure 4:**
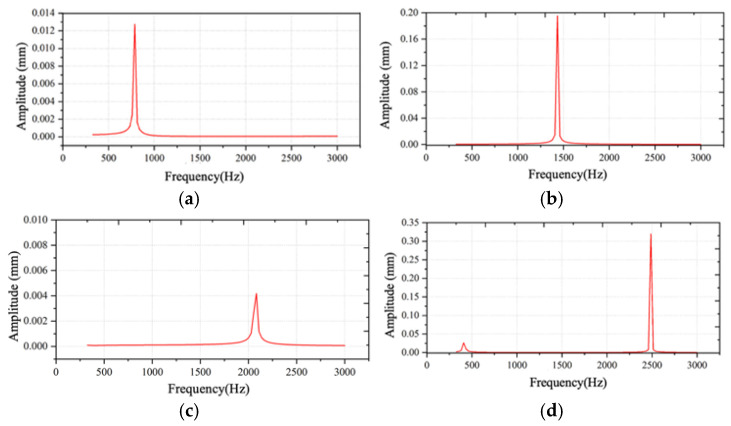
The A–f curve under of the cantilever under different structural parameter combinations: (**a**) *l* = 12.7, *n_h_* = 0.60; (**b**) *l* = 10.3, *n_h_* = 0.47; (**c**) *l* = 22.6, *n_h_* = 0.133; (**d**) *l* = 22.6, *n_h_* = 0.834.

Figure 4 presents representative amplitude–frequency (*A–f*) response curves of the cantilever under different structural parameter combinations. The results demonstrate that variations in cantilever length and piezoelectric placement significantly affect the resonance characteristics and vibration amplitude, leading to distinct dynamic response behaviors.

**Figure 5:**
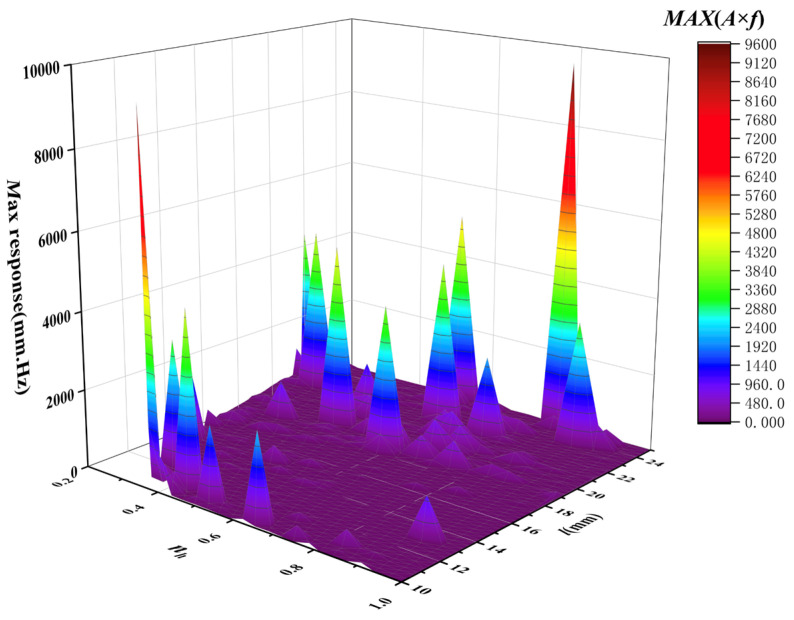
The maximum response of the cantilever under different structural parameter combinations.

To quantitatively assess the modulation performance of each structural configuration, the maximum value of the product A×f, denoted as max(A×f), was calculated from each frequency response curve. This metric effectively captures the combined contribution of vibration amplitude and modulation frequency, serves as a direct indicator of the modulation efficiency and sensor sensitivity. The resulting optimal response values for different structural configurations are summarized in Figure 5. As shown, the cantilever achieved its maximum response when l=24 mm and $n_{h}=0.8$, indicating that this parameter combination provides the highest modulation efficiency and is therefore most favorable for high-sensitivity sensing.

Under the optimized structural parameters, the maximum cantilever response occurred at a modulation frequency of 1725 Hz, corresponding to the resonant frequency of the cantilever. Typically, this frequency locates at the mechanical resonance frequency of the cantilever, which is sensitive to external disturbances. To mitigate potential measurement errors, the operating frequency is selected slightly below the resonant frequency. This ensures the cantilever’s vibration stability while maintaining sensor sensitivity. Accordingly, a modulation frequency of 1718 Hz was adopted in this study. The finalized mechanical parameters of the proposed sensor are summarized in Table 1.

**Figure 6:**
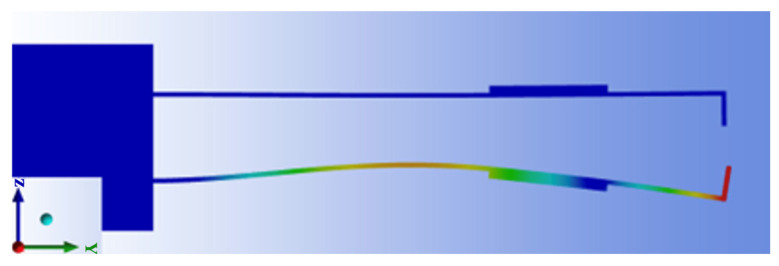
The vibration mode of the designed cantilever.

Figure 6 presents the vibration mode shape of the micro-vibrating cantilever under the optimized mechanical parameters. As illustrated, the cantilever operates in its second-order vibration mode, in which displacement primarily occurs within the *y*–*z* plane. This vibration mode offers high modulation efficiency for the sensing electrode. Moreover, the cantilever does not exhibit excessive elastic deformation under this configuration, ensuring reliable bonding between the piezoelectric ceramic and the cantilever. This structural stability is essential for maintaining long-term operational reliability and measurement consistency of the sensor.

To numerically validate the working mechanism of the proposed vibration-modulated electrostatic potential sensor, a three-dimensional finite-element model was developed based on the finite element method (FEM). The primary objective of this simulation was to verify that the proposed vibrating-capacitor configuration could effectively convert a static electrostatic potential, representative of DC power equipment surfaces, into a time-varying electrical signal suitable for measurement.

The simulation was implemented on the COMSOL Multiphysics 6.3 platform. To suppress artificial boundary effects, the sensor model was embedded in an air domain with dimensions 100 times larger than those of the sensor itself. The geometric models of the key components of the sensor, including the electrodes and shielding structures, were constructed based on the actual structural parameters to ensure modeling accuracy. A transient analysis was employed to capture the dynamic response of the sensor under vibration modulation. To emulate the high-frequency micro-vibration of the shielding electrode, a moving-mesh technique was adopted to impose a prescribed periodic displacement on the shielding structure. During the simulation, probe functions were defined to record the surface integrals of charge density and current density on the sensing electrode, allowing direct observation of the time-dependent electrical response.

**Figure 7:**
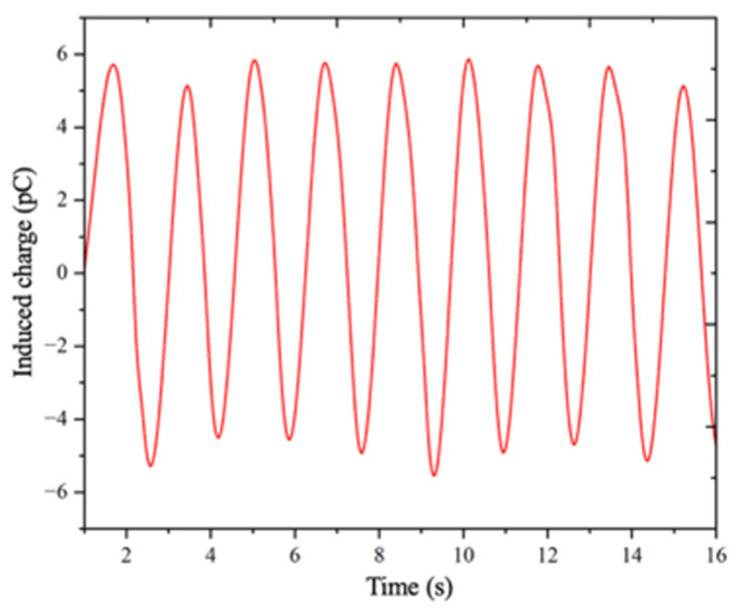
Simulation results of induced charge output from sensors under vibration modulation.

The simulation results, shown in Figure 7, indicate that the surface charge density on the sensing electrode varied sinusoidally with time under vibration modulation. Notably, the oscillation period of the induced charge was identical to the vibration period of the shielding electrode, confirming a direct correspondence between mechanical modulation and electrical response. Consequently, a periodic induced current was generated in the measurement circuit, which agrees well with the theoretical analysis. These results confirm that vibration-induced capacitance modulation transforms a static electrostatic potential into an alternating current signal. From both theoretical and numerical perspectives, the simulation outcomes validate the feasibility and effectiveness of the proposed sensor design for practical electrostatic potential sensing applications.

## 4. Experimental Results and Discussion

**Figure 8:**
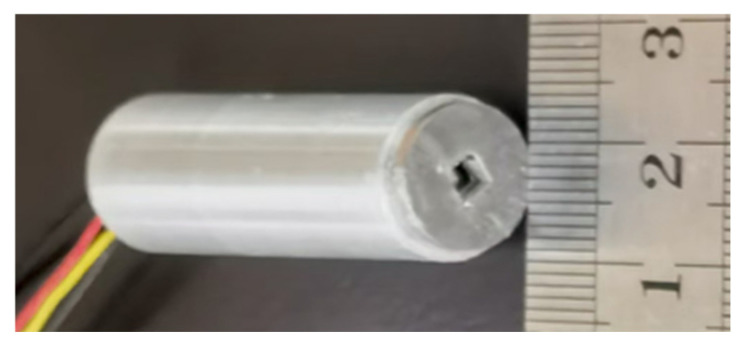
Photograph of the proposed electrostatic potential sensor.

Based on the theoretical analysis and structure optimization presented above, a cantilever micro-vibration-modulated electrostatic potential sensor was fabricated to experimentally validate the proposed sensing approach. Figure 8 shows a photograph of the developed sensor probe. The sensor is packaged in a cylindrical housing with an overall dimension of Φ12 mm × 35 mm, providing a compact and robust structure suitable for non-contact measurements. Here, the metal cylindrical shell can also suppress the coupling of external electromagnetic interference signals to the internal sensor circuits, thereby reducing environmental noise impact on the measurement results.

### 4.1. Experimental Platform and Signal Processing

**Figure 9:**
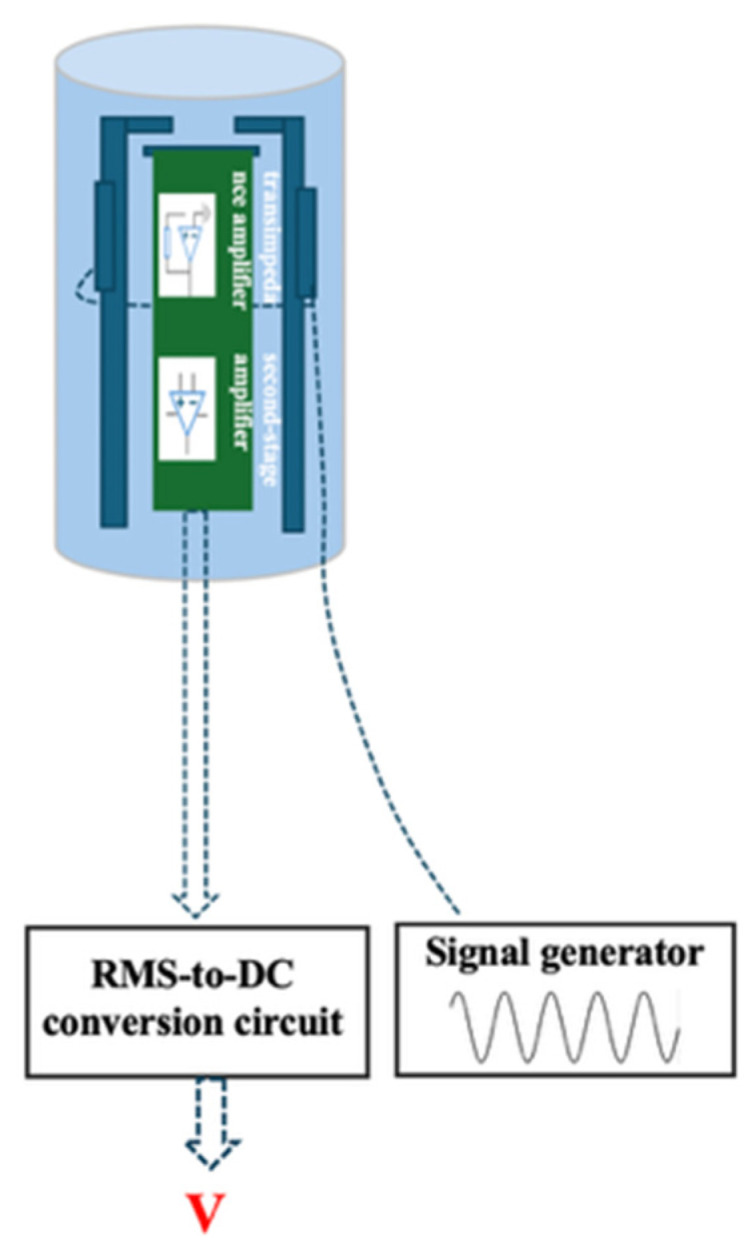
Signal demodulation system of the sensor.

The signal demodulation system of the sensor is illustrated in Figure 9. A signal generator provides a sinusoidal excitation signal with a frequency of 1718 Hz and an amplitude of 5 V, which is applied to the piezoelectric ceramic. Driven by the alternating electric field, the piezoelectric ceramic undergoes periodic micro-deformation, resulting in cantilever vibration and generation of picocoulomb-level charges on the sensing electrode.

The induced charge is first converted into a voltage signal by a transimpedance amplifier. Subsequently, a second-stage instrumentation amplifier performs differential amplification to suppress common-mode interference. The amplified signal is then filtered to improve the signal-to-noise ratio. Finally, an RMS detection circuit converts the alternating voltage signal into a DC voltage *V*, which is linearly related to the surface electrostatic potential of the measured object.

**Figure 10:**
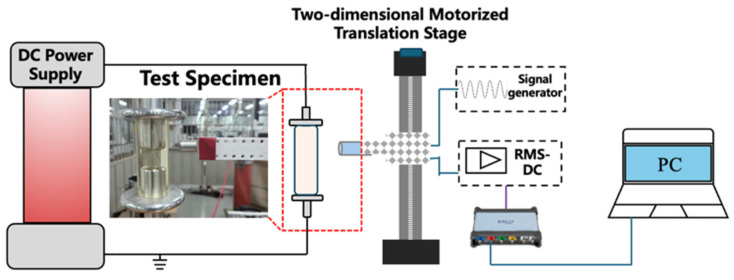
Experimental platform.

To evaluate the performance of the fabricated sensor, an experimental platform was established, as shown in Figure 10. A 200 kV DC high-voltage power supply (Shanghai Huidong Electrical Equipment Co., Ltd., Shanghai, China) with a voltage ripple factor below 0.5% was used as the excitation source. The electrostatic probe was mounted on a two-dimensional motorized translation stage with a travel range of ±200 mm and a positioning accuracy of ±100 μm. During measurements, the probe was scanned linearly along the axial direction of the insulator, from the grounded electrode toward the high-voltage electrode, at a constant speed of 200 mm/min. The sensor output signal, after RMS detection, was acquired by a data acquisition card and displayed in real-time on the host computer.

### 4.2. Sensors Calibration

To establish the quantitative relationship between the sensor output and the measured surface potential, calibration experiments were first conducted. During calibration, DC voltages ranging from 0 kV to 10 kV were applied to the test post insulator in increments of 1 kV. The developed electrostatic probe measured the potential at the high-voltage electrode position with a fixed working distance of 10 mm, and the corresponding sensor output amplitude was recorded. For each voltage level, measurements were repeated five times to ensure repeatability.

**Figure 11:**
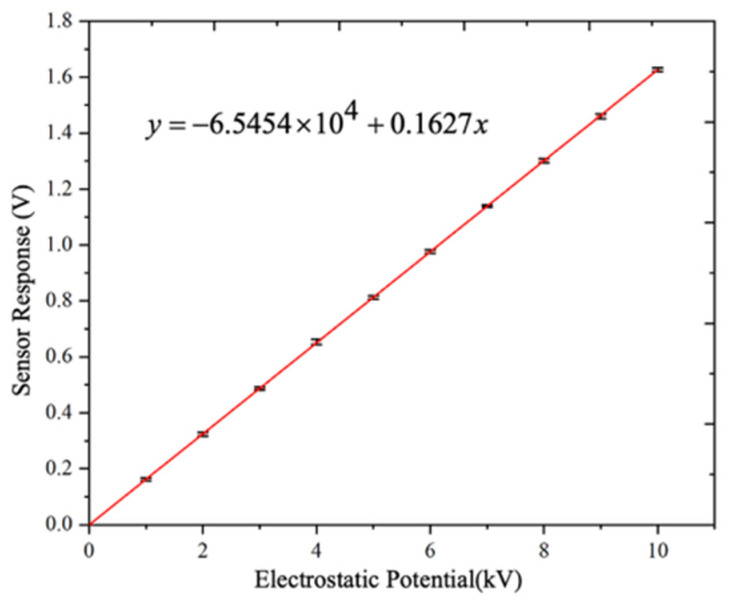
Calibration curve.

The resulting calibration curve relating the sensor output amplitude to the measured potential is shown in Figure 11, where the sensor output amplitude increased proportionally with the applied DC voltage over the entire calibration range from 1 to 10 kV, with a maximum measurement error of 0.92%. The calibration results clearly indicate that the output amplitude of the proposed sensor exhibited a strong linear relationship with the measured surface potential at a fixed working distance, with a linearity error of 0.47%. This linear behavior is consistent with the theoretical sensing model and confirms that under fixed modulation and distance conditions, the vibration-modulated induced current provides a reliable quantitative representation of the surface electrostatic potential.

Based on the calibration results, the relationship between the sensor output amplitude *V* and the measured potential *φ_E_* at a working distance of 10 mm can be expressed as:

(8) $$\varphi_{E}=\frac{V+6.545\times 10^{-4}}{0.1627}$$

Here, the equivalent surface potential resolution of the proposed sensor is estimated to be 30.7 V at noise level of t 5 mV.

According to the sensing principle described in Section 2, the sensor output amplitude is inherently dependent on the working distance between the probe and the measured surface. Therefore, to accurately reconstruct the surface potential under different measurement distances, a distance-dependent correction of the sensor response is required.

**Figure 12:**
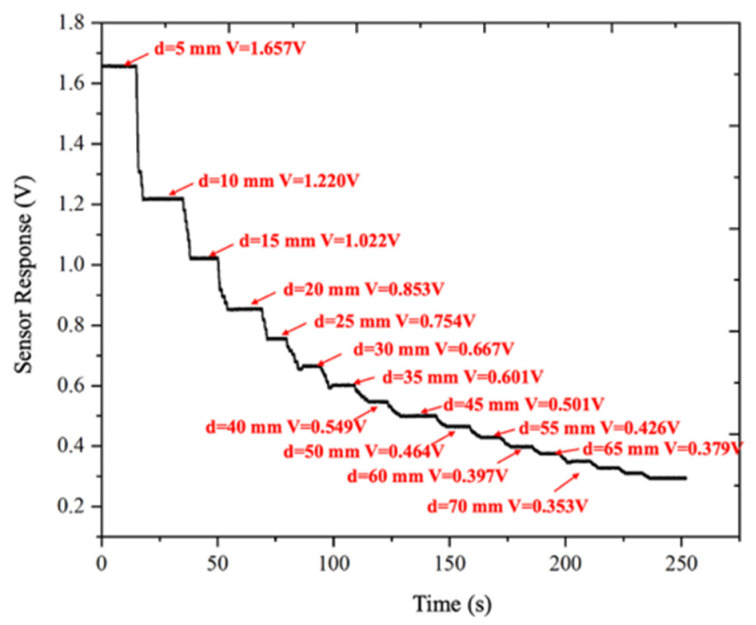
Sensor response under different working distances.

To this end, additional experiments were carried out under a constant applied DC voltage of 7.5 kV. The electrostatic probe measured the potential at the high-voltage electrode position while the working distance between the probe and the test specimen was varied using a two-dimensional motorized translation stage. The distance was increased from 5 mm to 70 mm in steps of 5 mm. At each distance, the signal was allowed to stabilize for at least 5 s before recording the sensor output amplitude. The experimental results are shown in Figure 12.

**Figure 13:**
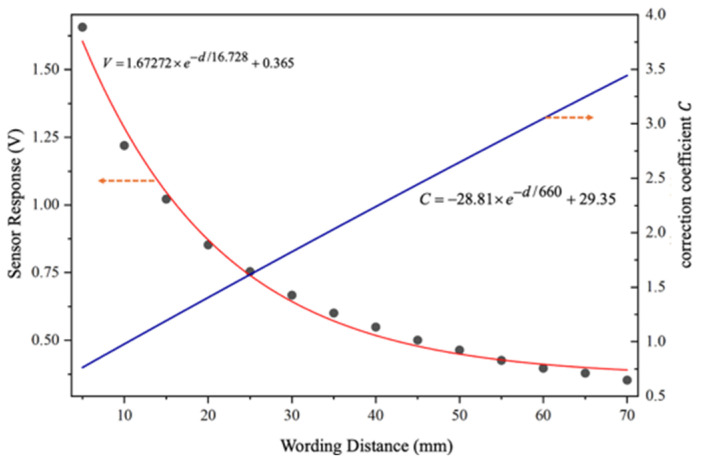
The correction coefficient vs. working distance.

The results indicate that for a fixed measured potential, the sensor output amplitude decreases monotonically with increasing working distance. Based on the experimental data, a correction curve describing the relationship between the sensor output amplitude *V* and the working distance *d* was established, as shown in Figure 13. Curve fitting revealed that the sensor output amplitude exhibits an exponential decay with respect to the working distance.

Taking the output amplitude at a working distance of 10 mm as the reference, the sensor output amplitude was normalized to obtain the working-distance correction coefficient *C*.

(9) $$C=-28.81\times e^{-d/660}+29.35$$

By combining Equations (8) and (9), the calibration equation relating the sensor output amplitude *V* to the measured potential at a working distance *d* can be expressed as

(10) $$\begin{array}{cc} \varphi_{E} & =6.15(V\cdot C+6.55\times 10^{-4})\\ & =(-177.18\cdot e^{-d/660}+180.52)\cdot V+4.28\times 10^{-3} \end{array}$$

This calibration–compensation coefficient not only compensates for the attenuation of the sensor output with increasing stand-off distance, but also provides a practical means to extend the measurable potential range while maintaining electrical safety. By appropriately selecting the measurement distance based on the electric field distribution of the tested object, the proposed sensor can be safely applied to higher-voltage scenarios without exceeding the breakdown limit of air.

### 4.3. Surface Potential Distribution Measurement of Insulator

To further verify the practical applicability of the proposed sensor, surface potential distribution measurements were performed on a post insulator. Based on the calibrated relationships described above, the electrostatic probe was mounted on the motorized translation stage and scanned linearly along the axial direction of the insulator, from the grounded electrode toward the high-voltage electrode, at a constant speed of 200 mm/min. The sensor output signal was processed through the RMS detection circuit, acquired by the data acquisition system, and displayed in real-time.

**Figure 14:**
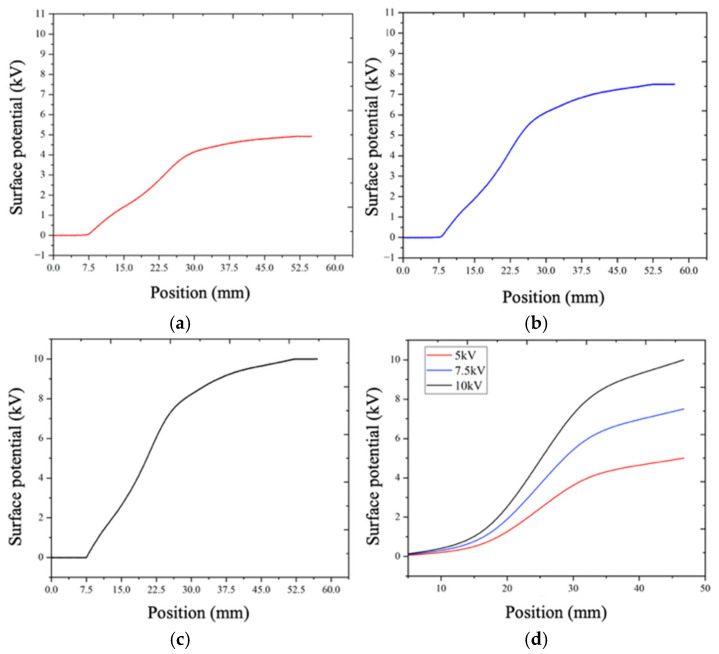
Surface potential measurements results: (**a**) 5 kV; (**b**) 7.5 kV; (**c**) 10 kV; (**d**) simulation results.

Surface potential measurements were performed under three applied DC voltages of 5 kV, 7.5 kV, and 10 kV. The resulting potential distribution curves are presented in Figure 14. As shown, the surface potential increased progressively from zero at the grounded electrode to the applied voltage at the high-voltage electrode.

A comparison between the experimentally measured potential distributions and the corresponding simulation results demonstrated close agreement in both trend and magnitude. This consistency confirms that the proposed sensor can accurately capture the surface potential distribution of insulating components, thereby validating its effectiveness and applicability for non-contact surface potential measurement in high-voltage power equipment.

## 5. Discussion

This paper presented a non-contact electrostatic potential sensor for surface potential measurement of insulating components in high-voltage DC power equipment. A cantilever-based micro-vibration modulation strategy driven by piezoelectric ceramics was employed to convert the static electrostatic potential into a measurable alternating current signal, effectively overcoming the charge decay and stability limitations associated with conventional DC electrostatic sensing techniques.

An electrostatic induction model was developed to describe the sensing mechanism and establish the quantitative relationship between sensor output and surface potential. Based on this analysis, the key structural parameters of the cantilever modulation system were optimized through multi-physics simulations, enabling efficient capacitance modulation, compact sensor dimensions, and reduced electric field disturbance. Finite-element simulations further confirmed that the proposed vibration-induced modulation can reliably convert a static electric field into a periodic charge and current response consistent with theoretical predictions.

Based on the optimized design, a prototype sensor and a dedicated experimental platform for electrostatic potential measurement of insulating components were developed. Calibration results demonstrate that the sensor achieved a maximum measurement error of 0.92% and a linearity of 0.47% over a measurement range of 1–10 kV. A working-distance correction model was also established to compensate for distance-dependent variations in sensor output, enabling flexible operation over practical stand-off distances. The proposed sensor was subsequently applied to measure the surface potential distribution of a post insulator under DC voltage. The measured potential profiles exhibited good agreement with simulation results, confirming the validity of the sensing principle and the reliability of the proposed method.

Compared with conventional field mill sensors, which prioritize ultra-high electric field sensitivity at the expense of sensor size and field disturbance, and Kelvin probe-based sensors, which require very short stand-off distances for high-resolution measurements, the proposed sensor offers a balanced solution tailored to high-voltage insulation diagnostics. By combining moderate potential resolution, compact size, adjustable working distance, and minimal electric field perturbation, the proposed sensor demonstrates clear application advantages for non-contact surface potential monitoring of high-voltage DC insulating components.

Practical high-voltage equipment often exhibits complex geometries and non-uniform electric field distributions, which may affect the spatial resolution of non-contact electrostatic measurements due to geometric and edge effects. These effects can be addressed by field inversion or reconstruction algorithms, which will be explored in future work. In addition, ambient humidity may influence the effective capacitance and measurement sensitivity; this impact can be mitigated through humidity-dependent calibration and real-time compensation.

## 6. Conclusions

This paper proposed a non-contact electrostatic potential sensor for surface potential measurement of insulating components in high-voltage DC power equipment, based on a piezoelectrically driven cantilever micro-vibration modulation mechanism. Theoretical modeling, multi-physics optimization, and experimental validation demonstrated that the sensor achieved stable potential measurement with a maximum error of 0.92% and a linearity of 0.47% over a 1–10 kV range while enabling flexible operation at practical stand-off distances. Compared with conventional field mill and Kelvin probe techniques, the proposed sensor provides a compact structure and reduced electric field disturbance, making it well-suited for high-voltage insulation diagnostics. These results indicate that the sensor offers a practical and scalable solution for non-contact surface potential monitoring of HVDC insulating components. The proposed micro-vibration-modulated electrostatic potential sensor provides a compact, sensitive, and reliable solution for surface potential monitoring in high-voltage DC insulation systems, offering promising prospects for condition assessment and electric field characterization of power equipment.

## Figures and Tables

**Table 1:** The mechanical parameter of the proposed sensor.

*l*/mm	*p*/mm	*h*/mm	*t*/mm	*a*/mm	*w*/mm
24	5	14.2	2	1.5	3

## Data Availability

Data are contained within the article and Supplementary Materials.

## Supplementary Materials

The following supporting information can be downloaded at: https://www.mdpi.com/article/10.3390/s26020362/s1, Table S1: Experimental Data.
